# Impact of Progress testing on the learning experiences of students in medicine, dentistry and dental therapy

**DOI:** 10.1186/s12909-018-1357-1

**Published:** 2018-11-09

**Authors:** Kamran Ali, Josephine Cockerill, Daniel Zahra, Christopher Tredwin, Colin Ferguson

**Affiliations:** 1 Faculty of Medicine and Dentistry, University of Plymouth, Plymouth, UK; 2 Peninsula Dental School, University of Plymouth, C523 Portland Square, Drake Circus, Plymouth, PL4 8AA UK

**Keywords:** Assessment, Education, Learning, Progress testing, Students, Undergraduate

## Abstract

**Aims:**

To investigate the impact of progress testing on the learning experiences of undergraduate students in three programs namely, medicine, dentistry and dental therapy.

**Methods:**

Participants were invited to respond to an online questionnaire to share their perceptions and experiences of progress testing. Responses were recorded anonymously, but data on their program, year of study, age, gender, and ethnicity were also captured on a voluntary basis.

**Results:**

A total of 167 participants completed the questionnaire yielding a response rate of 27.2% (*n* = 167). These included *96* BMBS students *(27.4%)*, *56* BDS students *(24.7%*), and 15 BScDTH students *(39.5%)*. A 3 -Program (BMBS, BDS, BScDTH) by 8-Topic (A-H) mixed analysis of variance (ANOVA) was conducted on the questionnaire responses. This revealed statistically significant main effects of Program and Topic, as well as a statistically significant interaction between the two (i.e. the pattern of topic differences was different across programs).

**Conclusions:**

Undergraduate students in medicine, dentistry, and dental therapy and hygiene regarded PT as a useful assessment to support their learning needs. However, in comparison to students in dentistry and dental therapy and hygiene, the perceptions of medical students were less positive in several aspects of PT.

## Introduction

Progress testing (PT) is now an established and accepted form of assessing applied knowledge in contemporary undergraduate medical curricula [1]. Students are examined throughout the program facilitating a longitudinal and comprehensive assessment of growth of knowledge [2]. The standard of questions is set at the level expected of a new graduate and students in all years of a program sit the same test simultaneously [1, 3]. Growth in applied knowledge is indexed by a steady increase in scores and enables reliable and valid decision-making about progression to the next stage of the program. PT is a feedback-oriented assessment and provides extensive opportunities for remediation of poorly-performing students; this allows students and their academic supervisors to identify areas of weakness and provide feedback to improve performance in successive years [4].

The rationale for the development and use of PT in undergraduate medical curricula is to minimize the traditional approaches to preparation adopted by the students for end-of-unit tests and may offer several advantages [5]. Whereas traditional end-of-module tests promote short-term, surface-level revision strategies, PT encourages students to acquire information throughout the duration of the module, breaking the link between learning and revision and reinforcing the spiral curriculum [6, 7]. Given that PT is aimed at testing the application of knowledge to real-life clinical situations, it may enhance students’ motivation for learning [8] and may help reduce stress associated with assessment by avoiding high stakes examinations [9].

Although these varied benefits of PT are widely reported in the literature, there are few published studies which explore the experiences and perceptions of the undergraduate medical students undertaking PT [10, 11]. The Faculty of Medicine and Dentistry, University of Plymouth uses PT for the assessment of knowledge in three different undergraduate programs and one postgraduate program: Bachelor of Medicine and Bachelor of Surgery (BMBS); Bachelor of Dental Surgery (BDS); Bachelor of Dental Therapy and Hygiene (BScDTH); and postgraduate diploma in Physician Associate (PA) studies. The undergraduate curricula for the BMBS, BDS and BScDTH programs were designed around a problem-based learning model with spiraling curriculum in successive years to allow review and repeated exposure to applied knowledge [3, 12, 13]. We have reported our experience in the development and use of PT in Medicine [4], Dentistry [14, 15] and Dental Therapy Programs [13]. To our knowledge, our institution is the first to use PT in BDS and BScDTH programs and we could not identify any published literature on the impact of PT on the learning experiences of students in these programs.

The aim of this study was to provide data allowing student perceptions of the impact of progress testing to be compared and contrasted across the three programs; BMBS, BDS, and BScDTH.

## Methods

Ethics approval for the study was obtained from the institutional Research Ethics Committee (Reference Number 16/17–695).

### Setting

The study was conducted at the Faculty of Medicine and Dentistry, University of Plymouth, United Kingdom (UK). The survey was open to responses throughout summer 2017.

### Study design, sample, and materials

Invitations to participate in the study were circulated by the Faculty Administrator to all current BMBS (*n* = 350), BDS (*n* = 227) and BScDTH (*n* = 38) students (*n* = 615) by e-mail. The invitation was accompanied by a participant information sheet detailing the purpose and scope of the study along with a URL to an online version of a previously validated questionnaire [9] designed to investigate the impact of progress testing on the learning experiences of students (Appendix). The questionnaire was hosted on Google Forms and all students were sent reminder two weeks following the initial invitation.

All participants completed online consent forms prior to completing the survey. Responses were recorded anonymously, but data on their program, year of study, age, gender and ethnicity were also captured on a voluntary basis. Whilst the number of and timestamps for the data submitted did not raise any concerns the risks of students submitting multiple forms and non-students submitting are to be acknowledged.

### Data analysis

Analyses were conducted using the R statistical environment for Windows https://www.r-project.org/.

Although the data are derived from ordinal level Likert-scale responses, and show some violations of parametric assumptions, previous work has shown that such data can be treated as interval for the purposes of analysis of variance (ANOVA) with minimal risk of Type I and Type II errors [16, 17], and it is largely treated as such in the medical education and social sciences literature. Furthermore, corrections for homogeneity of variance and equivalent non-parametric analyses conducted on the current data lead to the same statistical conclusions as ANOVA models; we therefore present the results of these familiar models to avoid the overall conclusions being confused by statistical nuance whilst acknowledging here the range of alternative analysis strategies for such data.

The questionnaire comprised 44 items, with 43 being scored on a Likert scale of one (strongly disagree) to five (strongly agree). Each of these items were allocated to one of eight ‘topics’ as shown in Table 1. The scoring of several items were reversed due to negative phrasing and these are indicated in Appendix, along with the group allocation and mean score by program for each item. Item 41 investigated the resources students use to prepare for a PT, offering five options, of which any number could be selected, along with a free text field. The data collected from this item has contributed to wider research [18].

**Table 1:** Group allocations of Items

ID	Topic	Item Numbers
A	Overall value of the Progress Test	1, 2, 3, 6, 8, 9, 10, 11, 12, 16, 17, 18, 24, 28, 39, 40
B	Preparation styles	15, 19, 23, 25, 26, 27, 42, 43, 44
C	Psychological impact	4, 5
D	Test behavior	37, 38
E	Test format/implementation	7
F	Value in assessing knowledge	29, 30, 31, 32, 33, 34, 35, 36
G	Value of test preparation	13, 14
H	Workload impact	20, 21, 22

## Results

The questionnaire was completed by 167 participants yielding a response rate of 27.2% (*n* = 167). These included *96* BMBS students *(27.4%)*, *56* BDS students *(24.7%)*, and 15 BScDTH students *(39.5%)*. The demographics (Program, Stage, Gender and Ethnicity) of the participants are detailed in Table 2. Whilst this study only considers Program as a factor, additional demographic data is reported to allow readers to assess the generalizability of our results to other cohorts and samples.

**Table 2:** Demographic characteristics of the participants

Factor	Level	N	%
Program	BMBS	96	57.5
	BDS	56	33.5
	BScDTH	15	9.0
Stage	Year 1	32	19.2
	Year 2	31	18.6
	Year 3	47	28.1
	Year 4^a^	41	24.5
	Year 5^a^	16	9.6
Gender	Male	66	39.5
	Female	99	59.3
	Not disclosed	2	1.2
Ethnicity	White	105	62.9
	Mixed: White and Black Caribbean	1	0.6
	Asian	42	25.1
	Mixed	3	1.8
	Black	8	4.8
	Arab	1	0.6
	Latin American	1	0.6
	Not disclosed	6	3.6

^a^BMBS and BDS students only (BScDTH is a three-year program)

### Topic scores by program

**Fig. 1:**
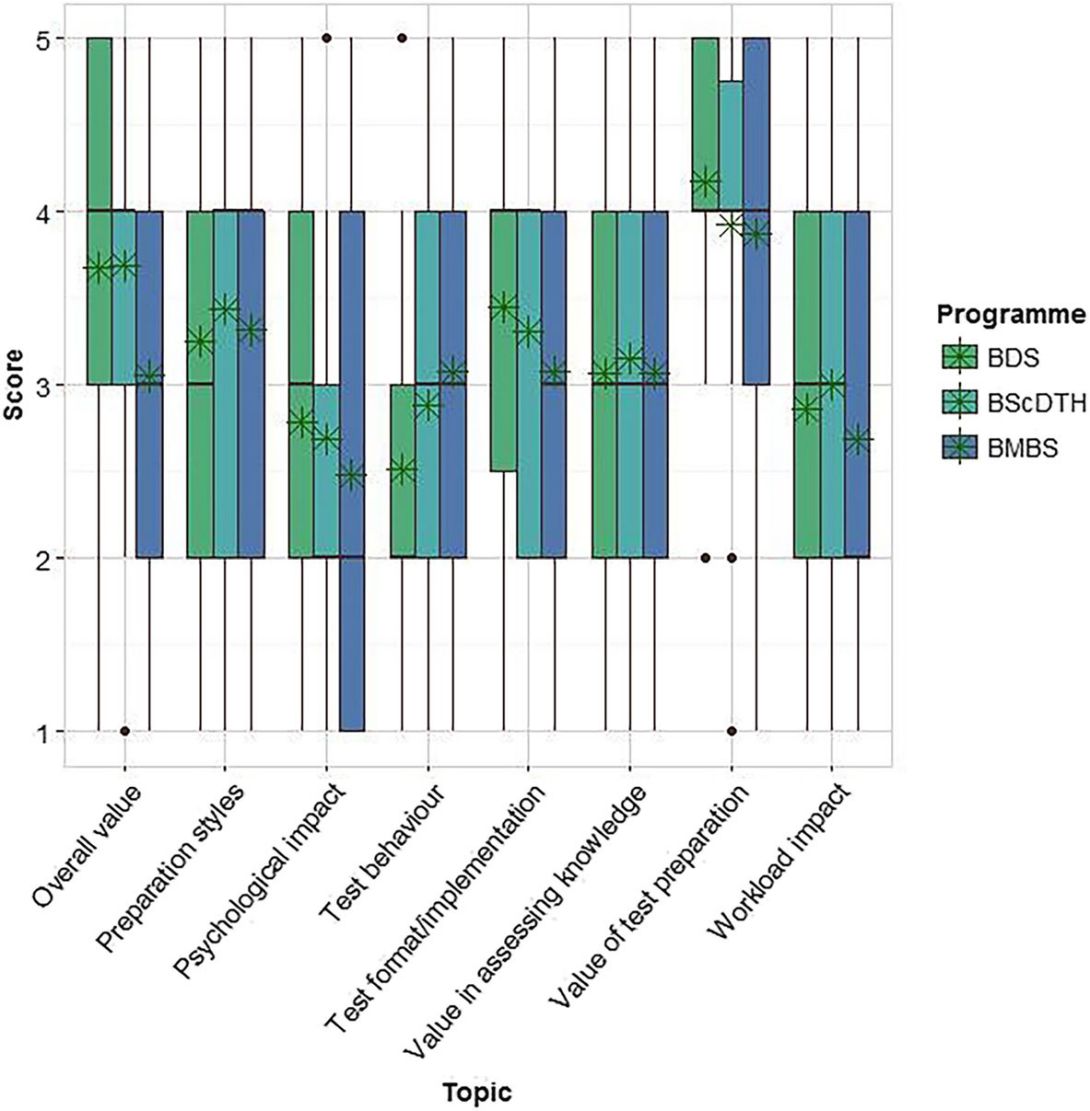
Boxplot of scores by topic, by program (program topic means indicated by stars)

Figure 1 shows the score distributions and observed mean scores (with reverse scoring applied) for each topic by program.

### Variation by item and program across topics

A 3 Program (BMBS, BDS, BScDTH) by 8 Topic (A-H) mixed analysis of variance (ANOVA) was conducted using the item level scores. This revealed statistically significant main effects of Program (*F*(2, 6682) = 33.20, *p* < 0.001) and Topic (*F*(7, 6682) = 44.38, *p* < 0.001), as well as a statistically significant interaction between the two (i.e. the pattern of topic differences was different across programs; *F*(14, 6682) = 8.01, *p* < 0.001).

### Variation by program and item within topics

The same ANOVA model was conducted on subsets of items by topic. Table 3 shows where there were statistically significant Program, Item, and Program-by-Item interactions for the scores within each topic. For example, the first row shows that students in different programs differed in their view of the overall value of the progress test, and their scores differed across items in this topic, but there was no program by item interaction for this group of questions (i.e. the same pattern of item differences was found across students in each programs).

**Table 3:** Student views by topic, with number of items, mean, standard deviation and *p*-values

Topic	Topic ID	Items (n)	Mean	StDev	*p*-value by Factor	Item	Prog: Item
					Program		
Overall value of Progress Test	A	16	3.310	1.287	< 0.001	0.019	0.155
Preparation styles	B	9	3.307	1.252	0.350	0.834	0.016
Psychological impact	C	2	2.596	1.241	0.058	0.733	0.023
Test behavior	D	2	2.875	1.229	< 0.001	0.515	0.009
Test format/implementation	E	1	3.218	1.209	0.199	–	–
Value in assessing knowledge	F	8	3.078	1.217	0.805	0.018	0.201
Value of test preparation	G	2	3.978	1.101	0.104	0.584	0.940
Workload impact	H	3	2.769	1.303	0.104	< 0.001	0.409

Of most interest to this project are the differences between Programs and Programs by Item. The former identifies differences in attitudes to progress testing between students on different programs, the latter identifies where there may be differential responding to items in each topic across students in different programs. As can be seen in Table 3, a statistically significant effect of Program was found in Topics A (overall value of Progress Test) and D (Test Behavior), and a statistically significant interaction between Program and Item was found in Topics B (Preparation Styles), C (Psychological Impact) and D (Test Behavior).

Although significant differences were found between individual items within topics, these are of less value to the research questions and were not subjected to post-hoc Tables 4, 5, 6 and 7 provide the results of post-hoc analysis by topic, showing the estimate (the difference between the means), the standard error associated with the difference between the means, the *t*-test, and the *p*-value associated with these analyses.

**Table 4:** Test of between Program effects (Tukey’s HSD) for Items in Topic A

Item	Question	Interaction	Estimate	Std. Error	t-value	p-value
1	The Progress Test is a useful form of examination.	BMBS-BDS	−0.92	0.196	−4.707	0.003
		BScDTH-BDS	−0.333	0.348	−0.958	> 0.999
		BScDTH-BMBS	0.587	0.332	1.769	> 0.999
2	The Progress Test is not a fair test.	BMBS-BDS	−1.052	0.196	−5.38	< 0.001
		BScDTH-BDS	−0.439	0.348	−1.261	> 0.999
		BScDTH-BMBS	0.613	0.332	1.847	> 0.999
3	The Progress Test is a waste of time.	BMBS-BDS	−0.558	0.196	−2.852	> 0.999
		BScDTH-BDS	−0.24	0.348	−0.689	> 0.999
		BScDTH-BMBS	0.318	0.332	0.958	> 0.999
6	The Progress Test questions are too clinically based to be applicable to students in early years.	BMBS-BDS	−1.639	0.196	−8.381	< 0.001
		BScDTH-BDS	0.121	0.348	0.347	> 0.999
		BScDTH-BMBS	1.759	0.332	5.302	< 0.001
8	The Progress Test is a good way to examine what we learn day to day on the course.	BMBS-BDS	−1.559	0.196	−7.973	< 0.001
		BScDTH-BDS	−0.059	0.348	−0.169	> 0.999
		BScDTH-BMBS	1.5	0.332	4.521	0.007
9	The Progress Test has little bearing on whether I go on to pass or fail the year.	BMBS-BDS	0.156	0.196	0.799	> 0.999
		BScDTH-BDS	−0.291	0.348	−0.837	> 0.999
		BScDTH-BMBS	−0.447	0.332	−1.348	> 0.999
10	I would be encouraged to work harder for an exam that just tested areas we had already covered.	BMBS-BDS	1.063	0.196	5.439	< 0.001
		BScDTH-BDS	0.041	0.348	0.117	> 0.999
		BScDTH-BMBS	−1.023	0.332	−3.082	> 0.999
11	We get enough feedback from the Progress Test to let us know how we are getting on in our course.	BMBS-BDS	−0.306	0.196	−1.563	> 0.999
		BScDTH-BDS	0.258	0.348	0.741	> 0.999
		BScDTH-BMBS	0.564	0.332	1.698	> 0.999
12	The Progress Test is good preparation for my future career	BMBS-BDS	−0.528	0.196	−2.7	> 0.999
		BScDTH-BDS	0.157	0.348	0.451	> 0.999
		BScDTH-BMBS	0.685	0.332	2.064	> 0.999
16	The Progress Test does not reward those who have worked hard throughout the year.	BMBS-BDS	−0.603	0.196	−3.086	> 0.999
		BScDTH-BDS	−0.472	0.348	−1.357	> 0.999
		BScDTH-BMBS	0.131	0.332	0.396	> 0.999
17	The Progress Test motivates me to work hard all year.	BMBS-BDS	−0.494	0.196	−2.527	> 0.999
		BScDTH-BDS	0.207	0.348	0.594	> 0.999
		BScDTH-BMBS	0.701	0.332	2.112	> 0.999
18	I do well in the Progress Test because I work hard throughout the year.	BMBS-BDS	−0.329	0.196	−1.682	> 0.999
		BScDTH-BDS	0.264	0.348	0.759	> 0.999
		BScDTH-BMBS	0.593	0.332	1.787	> 0.999
24	Preparing for other assessments helps me prepare for the Progress Test.	BMBS-BDS	−0.496	0.196	−2.539	> 0.999
		BScDTH-BDS	0.205	0.348	0.59	> 0.999
		BScDTH-BMBS	0.702	0.332	2.114	> 0.999
28	Patient contact in the early years is helpful preparation for the Progress Test.	BMBS-BDS	−0.752	0.196	−3.847	0.135
		BScDTH-BDS	0.635	0.348	1.825	> 0.999
		BScDTH-BMBS	1.387	0.332	4.18	0.033
39	I think the Progress Test is a good way of assessing a EBL/PBL based curriculum.	BMBS-BDS	−1.244	0.196	−6.365	< 0.001
		BScDTH-BDS	0.087	0.348	0.251	> 0.999
		BScDTH-BMBS	1.332	0.332	4.014	0.067
40	The Progress Test helps me apply my knowledge to clinical situations.	BMBS-BDS	−0.696	0.196	−3.559	0.419
		BScDTH-BDS	0.133	0.348	0.381	> 0.999
		BScDTH-BMBS	0.829	0.332	2.497	> 0.999

**Table 5:** Test of between Program effects (Tukey’s HSD) for Items in Topic B

Item	Question	Interaction	Estimate	Std. Error	t-value	p-value
15	Most of my preparation for the Progress Test is done at the last minute.	BMBS-BDS	0.334	0.201	1.662	> 0.999
		BScDTH-BDS	−0.449	0.358	−1.257	> 0.999
		BScDTH-BMBS	−0.783	0.341	−2.298	> 0.999
19	Last minute preparation helps me improve my grade on the Progress Test	BMBS-BDS	0.311	0.201	1.546	> 0.999
		BScDTH-BDS	−0.465	0.358	−1.299	> 0.999
		BScDTH-BMBS	−0.775	0.341	−2.273	> 0.999
23	I prepare for the Progress Test alone.	BMBS-BDS	− 0.103	0.201	−0.512	> 0.999
		BScDTH-BDS	−0.122	0.358	−0.342	> 0.999
		BScDTH-BMBS	−0.019	0.341	−0.056	> 0.999
25	In preparation for the Progress Test it is better to try and prepare a couple of topics in depth than to try and learn everything.	BMBS-BDS	−0.258	0.201	−1.285	> 0.999
		BScDTH-BDS	0.582	0.358	1.628	> 0.999
		BScDTH-BMBS	0.84	0.341	2.464	> 0.999
26	I find it useful to prepare in pairs or groups.	BMBS-BDS	0.12	0.201	0.595	> 0.999
		BScDTH-BDS	0.615	0.358	1.721	> 0.999
		BScDTH-BMBS	0.496	0.341	1.454	> 0.999
27	I think spending time working in clinical environments is a good way to prepare for the Progress Test.	BMBS-BDS	−0.111	0.201	− 0.553	> 0.999
		BScDTH-BDS	0.537	0.358	1.502	> 0.999
		BScDTH-BMBS	0.648	0.341	1.9	> 0.999
42	Textbooks are not the best source for preparation.	BMBS-BDS	0.947	0.201	4.712	0.001
		BScDTH-BDS	−0.202	0.358	−0.565	> 0.999
		BScDTH-BMBS	−1.149	0.341	−3.369	0.265
43	My preparation for the Progress Test would be improved if I made better notes.	BMBS-BDS	−0.644	0.201	−3.206	0.473
		BScDTH-BDS	0.264	0.358	0.738	> 0.999
		BScDTH-BMBS	0.908	0.341	2.663	> 0.999
44	Doing example MCQs is the most effective way to prepare.	BMBS-BDS	0.638	0.201	3.175	0.526
		BScDTH-BDS	−0.03	0.358	−0.084	> 0.999
		BScDTH-BMBS	−0.668	0.341	−1.959	> 0.999

**Table 6:** Test of between Program effects (Tukey’s HSD) for Items in Topic C

Item	Question	Interaction	Estimate	StdError	t	p
4	Not knowing what will come up in the Progress Test makes me feel anxious.	BMBS-BDS	0.06	0.213	0.282	> 0.999
		BScDTH-BDS	−0.297	0.378	−0.785	> 0.999
		BScDTH-BMBS	−0.357	0.361	−0.99	> 0.999
5	It is disheartening to sit an exam with questions to which I know so few of the answers.	BMBS-BDS	−0.672	0.213	−3.161	0.024
		BScDTH-BDS	0.113	0.378	0.299	> 0.999
		BScDTH-BMBS	0.785	0.361	2.176	0.443

**Table 7:** Test of between Program effects (Tukey’s HSD) for Items in Topic D

Item	Question	Interaction	Estimate	Std. Error	t-value	p-value
37	I guess the answers to most of the questions in the Progress Test.	BMBS-BDS	0.965	0.2	4.815	< 0.001
		BScDTH-BDS	0.173	0.357	0.486	> 0.999
		BScDTH-BMBS	−0.792	0.34	−2.327	0.299
38	I think it is more honest to state “I don’t know” than it is to guess the answer.	BMBS-BDS	0.167	0.2	0.835	> 0.999
		BScDTH-BDS	0.576	0.357	1.615	> 0.999
		BScDTH-BMBS	0.409	0.34	1.202	> 0.999

### Post hoc analysis by topic

#### Student views on the overall value of the Progress test (topic a)

Post-hoc analysis of the Program differences in the overall value of Progress Tests revealed that there were significant differences for seven of the 16 items within this topic, with the differences being between the BMBS and BDS and/or the BMBS and BScDTH program, as highlighted in Table 4. The specific differences are outlined below; where a comparison between two or more groups is omitted, it was not found to be statistically significant.

Across the three programs students agreed that the PT was a useful form of assessment, with the BDS students agreeing significantly more with this statement (Item 1; BDS mean = 4.33 versus 4.00 and 3.41 for BScDTH and BMBS respectively). BDS (M = 3.82) and BMBS (M = 2.77) student responses differed significantly in their scores for the statement ‘The Progress Test is not a fair test’, with the BMBS students agreeing more strongly that it is not a fair test (Item 2). BMBS students were significantly more inclined than both the BDS and BScDTH students to agree that the PT questions are too clinically based to be applicable to students in the early years (Item 6; means of 3.85, 3.73, and 2.09 for BScDTH, BDS, and BMBS respectively).

BDS (M = 4.06) and BScDTH (M = 4.00) students agreed that the PT is a good way to examine what they learn day to day on the course, with the BMBS students (M = 2.09) agreeing significantly less with this statement (Item 8). BMBS students agreed significantly more than the BDS students that they would be encouraged to work harder for an examination that just tested areas they had already covered (Item 10; means of 3.95 and 2.88 for BMBS and BDS respectively). BScDTH students agreed significantly more strongly than the BMBS students that patient contact in the early years is helpful preparation for the PT (Item 28; means of 4.62 and 2.21 for BScDTH and BMBS respectively). There was no strong positive agreement that the PT is a good way of assessing an EBL/PBL based curriculum, with BMBS students (M = 2.21) agreeing significantly less than the BDS students (M = 3.45, Item 39).

#### Preparation styles (topic B)

Of the nine items in this topic, program scores only differed within item 42, with only the difference between the BMBS (M = 3.46) and BDS (M = 2.51) programs reaching statistical significance (Tukey’s HSD, *p* = 0.024). BMBS students agreed significantly more strongly than the BDS students that textbooks are not the best source for preparation as shown in Table 5.

#### Psychological impact of the Progress test (topic C)

Of the two items in this topic, 4 and 5, program scores only differed within item 5, with only the difference between the BMBS (M = 3.12) and BDS (M = 2.45) programs reaching statistical significance (Tukey’s HSD, *p* = 0.024). BMBS students agreed significantly more strongly than the BDS students that it was disheartening to sit an exam with questions to which they knew so few of the answers (Item 5, reverse scored). These results are summarized in Table 6.

#### Test behavior (topic D)

Of the two items in this topic, 37 and 38, program scores only differed within Item 37, with only the difference between BDS and BMBS reaching statistical significance (Tukey’s HSD, *p* < 0.001). BMBS students were significantly more likely than BDS students to agree with the statement that they guessed the answers to most of the items in the PT (Item 37; means of 2.95, 2.15, and 1.98 for BMBS, BScDTH, and BDS respectively). These results are depicted in Table 7.

## Discussion

This is the first study exploring undergraduate experiences of PT across three different programs in healthcare education. Several advantages of PT over traditional assessment methods have been highlighted in the literature [19–21]. However, most published studies focus on the philosophy, format, and metrics of PT along with perspectives by the experts [3, 22]. Notwithstanding the need to ensure that assessments are valid, reliable, and feasible, it is crucial that the assessment methods are acceptable to the stakeholders [6]. Given that students are the key stakeholders in the assessments used in educational programs, it is imperative to gauge their perceptions and experiences to inform the future development of assessment methods. Previous studies have reported that educators and students may sometimes be at odds about the usefulness of curriculum interventions and assessments [23]. Although a small number of studies have reported the views and experiences of undergraduate medical students on PT [10, 11, 24], there are no previous studies involving undergraduate students in Dentistry and Dental Therapy and Hygiene.

Overall, students across all programs were positive about the value of PT as a useful assessment to support their learning in their respective domains of study. However, BMBS students were less positive about the clinical context of PT compared to BDS and BScDTH students. One possible explanation for this variation is that dental students get more structured clinical exposure in early years at our institution [25]. Dentistry is a unique pedagogical experience and training in dentistry involves performing irreversible operative procedures on patients under supervision [26]. BDS and BScDTH students at Plymouth University start treating patients towards the end of Year 1 of their respective programs and this may account for their ability to apply knowledge to clinical situations in early years of the program. Differences in the level of clinical exposure in early years may also account for lower scores reported by medical students with regards to the impact of enquiry-based learning and prior learning on their preparation and performance on PT. It has been reported that early clinical exposure translates into improved perceptions about the usefulness of PT and consequentially students are more likely to use a deep learning approach [10].

Another possible explanation for lower scores reported by medical students may be related to differences in the format and standard setting of PT for the BMBS program compared to those for the BDS and BScDTH programs at our institution. Firstly, the BMBS students sit 125-item single-best-answer multiple choice question assessments four times each academic year. On the other hand, PT for the BDS and BScDTH programs are homogenous in regard to the format, frequency and standard setting. BDS and BScDTH students sit 100-item single-best-answer multiple-choice assessments once per term (three times annually). Moreover, the final-year BMBS assessments are criterion-referenced with a pass-fail outcome, whereas earlier years are norm-referenced against set-proportion categorical grading. The BDS and DTH programs however are standard set using a combined Angoff-Hofstee procedure to generate a cut-score, around which categorical grade boundaries are constructed perceptions of discrimination between programs [27, 28]. Our findings are supported by previous studies on medical students which show that the format and specific details of how PT is conducted has an impact on student learning [10].

Assessments are generally reported to be stressful for students [29]. Overall, the students reported mixed perceptions regarding the psychological impact of PT with, medical students being less positive. As explained earlier, these variations between medical and dental students may be attributed to the differences in the curriculum design, clinical exposure and format of PT, as well as variation in progression rules. Another study on medical students has reported that student stress associated with PT may be related to a general lack of understanding about the purpose of PT and struggles in developing a strategy to answer questions. Nevertheless, attitudes are likely to improve as the students progress through the program, developing strategies for using their time effectively in the assessment and an understanding of the underlying philosophy of PT [11].

One of the limitations of this study is a low response rate especially for the BMBS program. Moreover, the data was collected from a single institution. However, the authors have no reason to suspect the sample differs in any critical respect to the wider population of students in our institution or healthcare education more widely. However, future work may benefit from sampling across multiple sites to increase sample size and supplementing the quantitative measures with open-ended questions and qualitative exploration of differing perceptions across programs. Another potentially fruitful avenue of investigation would be to further our understanding of the views of academic staff, and explore how these may be reflected by, or otherwise influence, student perceptions. Where the current results begin to shed light on student perceptions of progress testing and its impact on their learning, these additional dimensions would further develop our understanding of the impact of progress testing across different domains of healthcare education.

## Conclusion

Undergraduate students in medicine, dentistry, and dental therapy and hygiene regarded PT as a useful assessment to support their learning needs. However, in comparison to students in dentistry and dental therapy and hygiene, the perceptions of medical students were less positive in several aspects of PT. These variations may, in part, be attributed to differences in clinical exposure in early years and test standardization. Further research with a range of stakeholders is required to establish the causes of these differences and develop our understanding of the perceived value and impact of PT on the learning experiences of healthcare students in undergraduate programs.

## Appendix

**Table 8:** Item number, question, negative score status, topic ID and mean score by program

Item	Question	Neg.	Topic ID	BMBS mean	BDS mean	BScDTH mean
1	The Progress Test is a useful form of examination.	No	A	4.00	3.41	4.32
2	The Progress Test is not a fair test.	Yes	A	3.40	2.74	3.80
3	The Progress Test is a waste of time.	Yes	A	4.20	3.94	4.45
4	Not knowing what will come up in the Progress Test makes me feel anxious.	Yes	C	2.20	2.50	2.39
5	It is disheartening to sit an exam with questions to which I know so few of the answers.	Yes	C	3.07	2.48	3.02
6	The Progress Test questions are too clinically based to be applicable to students in early years.	Yes	A	3.87	2.09	3.65
7	I get too tired by the end of the Progress Test to perform well.	Yes	E	3.27	3.07	3.52
8	The Progress Test is a good way to examine what we learn day to day on the course.	No	A	3.93	2.49	4.02
9	The Progress Test has little bearing on whether I go on to pass or fail the year.	Yes	A	3.87	4.32	4.17
10	I would be encouraged to work harder for an exam that just tested areas we had already covered.	No	A	2.93	3.97	2.91
11	We get enough feedback from the Progress Test to let us know how we are getting on in our course.	No	A	2.87	2.29	2.63
12	The Progress Test is good preparation for my future career	No	A	3.87	3.31	3.82
13	I think preparing for the Progress Test is important.	No	G	4.00	3.91	4.20
14	There is no point preparing for the Progress Test.	Yes	G	3.87	3.84	4.20
15	Most of my preparation for the Progress Test is done at the last minute.	Yes	B	1.93	2.75	2.32
16	The Progress Test does not reward those who have worked hard throughout the year.	Yes	A	2.93	2.99	3.50
17	The Progress Test motivates me to work hard all year.	No	A	3.47	2.73	3.27
18	I do well in the Progress Test because I work hard throughout the year.	No	A	3.53	2.96	3.32
19	Last minute preparation helps me improve my grade on the Progress Test.	No	B	2.67	3.49	3.11
20	I do not have time to prepare for the Progress Test.	Yes	H	3.27	3.26	3.70
21	I think we should be given time in the timetable to prepare for the Progress Test.	No	H	3.60	2.98	3.07
22	Six hours is sufficient preparation for the Progress Test.	No	H	2.13	1.82	1.88
23	I prepare for the Progress Test alone.	No	B	3.27	3.22	3.30
24	Preparing for other assessments helps me prepare for the Progress Test.	No	A	3.53	2.84	3.30
25	In preparation for the Progress Test it is better to try and prepare a couple of topics in depth than to try and learn everything.	No	B	3.13	2.48	2.73
26	I find it useful to prepare in pairs or groups.	No	B	3.40	3.17	3.05
27	I think spending time working in clinical environments is a good way to prepare for the Progress Test.	No	B	4.60	3.96	4.07
28	Patient contact in the early years is helpful preparation for the Progress Test.	No	A	4.60	3.23	4.00
29	The Progress Test is more about pattern recognition than knowledge and understanding.	No	F	2.2	3.68	2.13
30	The Progress Test helps me improve my knowledge.	No	F	3.87	3.11	4.20
31	I am able to monitor how my knowledge is improving through the Progress Test.	No	F	3.73	3.29	3.82
32	Other assessments help me improve my knowledge more than the Progress Test does.	No	F	2.73	3.54	2.50
33	Students with poor knowledge can still pass the Progress Test.	Yes	F	2.93	2.05	2.88
34	The Progress Test is a good way to assess what we learn in clinical environments	No	F	4.13	3.18	3.64
35	The Progress Test does not allow me to show the knowledge I have.	Yes	F	2.87	2.38	2.93
36	My performance in the Progress Test reflects luck more than knowledge.	No	F	2.53	3.38	2.54
37	I guess the answers to most of the questions in the Progress Test.	No	D	2.13	2.96	1.95
38	I think it is more honest to state “I don’t know” than it is to guess the answer.	No	D	3.60	3.23	3.00
39	I think the Progress Test is a good way of assessing a EBL/PBL based curriculum.	No	A	3.53	2.19	3.36
40	The Progress Test helps me apply my knowledge to clinical situations.	No	A	4.20	3.43	4.09
42	Textbooks are not the best source for preparation.	No	B	2.20	3.45	2.54
43	My preparation for the Progress Test would be improved if I made better notes.	No	B	3.47	2.69	3.21
44	Doing example MCQs is the most effective way to prepare.	No	B	3.60	4.21	3.61

## Abbreviations

ANOVA: Analysis of Variance
BDS: Bachelor of Dental Surgery
BMBS: Bachelor of Medicine and Bachelor of Surgery
BScDTH: Bachelor of Science Dental Therapy and Hygiene
PA: Physician Associate
PT: Progress Testing
UK: United Kingdom
